# Supplementary material for: Reforming teaching methods by integrating dental theory with clinical practice for dental students
Source: PeerJ. 2020 Feb 7;8:e8477. doi: 10.7717/peerj.8477 (PMC7008813; doi:10.7717/peerj.8477)
Supplement: Supplemental Information 1 [file peerj-08-8477-s001.docx]

| Items and scoring rules | Scores  (Max points) | Scores |
| --- | --- | --- |
| 1. Whether the appearance of the students meets the hygiene requirements. For example, whether the hat, mask, and glove are worn correctly. | 5 |  |
| 2. Whether the students' attitude is pleasant when they face patients, and whether the speed of their speech is appropriate. | 5 |  |
| 3. Whether the inquiry is detailed, whether the purpose and requirements of patients are understood, and whether the patients' urgent issues to be addressed and comprehensive history, including the history, drug allergies, etc., are collected. | 15 |  |
| 4. When conducting oral preliminary examination, whether compliance with aseptic conditions is satisfactory, whether the mouth pulling action is gentle, and whether the chair position is appropriate. | 5 |  |
| 5. Whether the oral examination is complete, comprehensive, includes a related repair inspection, includes the abutments, the gaps of missing teeth, the alveolar ridge and mucosa, occlusion, etc., and examination of other dental, periodontal, and mucosal conditions | 15 |  |
| 6. Whether the auxiliary check is reasonable and comprehensive, whether the diagnosis of oral diseases is accurate and complete, and whether a reasonable differential diagnosis is conducted. | 10 |  |
| 7. Whether the preliminary diagnosis is correct, whether the explanation of the oral condition is sufficiently detailed, and whether several possible treatment plans are developed, including any necessary collaborative treatments involving other departments. | 15 |  |
| 8. Whether a reasonable treatment plan has been determined and described in detail to the patients, including the desired treatment time, costs, possible problems, etc. | 15 |  |
| 9. Whether the case history record is comprehensive and standardized. | 5 |  |
| 10. Whether the patients' recognition and satisfaction are received. | 10 |  |
| Total | 100 |  |

Score sheet for clinical practice

| Question | obviously | a little | hardly |
| --- | --- | --- | --- |
| Have your ability in independently searching literatures increased? |  |  |  |
| Have your comprehensive and logical analysis skills increased? |  |  |  |
| Have your teamwork ability increased? |  |  |  |
| Have your curiosity and exploratory desire of professional knowledge risen? |  |  |  |

Questionnaire on student abilities and skills changes after clinical simulation of PBL training

| Question | obviously | a little | hardly |
| --- | --- | --- | --- |
| Have your ability in grasping indications for repair increased? |  |  |  |
| Have your ability in correctly diagnosing the diseases increased? |  |  |  |
| Have your ability in developing the treatment plans increased? |  |  |  |
| Have your ability in quickly and accurately grasping the patient’s condition increased? |  |  |  |
| Have your ability in communicating with patients increased? |  |  |  |
| Have your understanding patients improved? |  |  |  |

Questionnaire on the treatment of dental disease and changes in communication skills with patients after clinical simulation of PBL training

Questionnaire on students' perceptions of teaching methods.

| Question | Yes | No |
| --- | --- | --- |
| Do you like this teaching method? |  |  |
| Is this method efficient for the use of time? |  |  |
| Does this teaching method help to enhance your problem-solving confidence? |  |  |
| Does this teaching method help to increase your interest in prosthodontics? |  |  |
| Does this teaching method help you with understanding the theoretical knowledge of prosthodontics? |  |  |
| Would you introduce this teaching method to others? |  |  |

Questionnaire about students' answers to questions: How do you discover the value of this teaching method compared to traditional teaching?

| Question | Much more | A little more | Almost the same |
| --- | --- | --- | --- |
| How did you find the value of this teaching method, as compared with that of the traditional teaching? |  |  |  |
